# The global trends and regional differences in incidence of Zika virus infection and implications for Zika virus infection prevention

**DOI:** 10.1371/journal.pntd.0010812

**Published:** 2022-10-21

**Authors:** Zirui Guo, Wenzhan Jing, Jue Liu, Min Liu

**Affiliations:** Department of Epidemiology and Biostatistics, School of Public Health, Peking University, Beijing, China; Army Hospital Research and Referral, INDIA

## Abstract

**Background:**

Zika virus (ZIKV) infection has potential result in severe birth effects. An improved understanding of global trend and regional differences is needed.

**Methods:**

Annual ZIKV infection episodes and incidence rates were collected from Global Burden of Disease Study 2019. Episodes changes and estimated annual percentage changes (EAPCs) of age-standardized incidence rate (ASR) were calculated. Top passenger airport-pairs were obtained from the International Air Transport Association to understand places susceptible to imported ZIKV cases.

**Results:**

Globally, the ASR increased by an average of 72.85% (95%CI: 16.47% to 156.53%) per year from 2011 to 2015 and subsequently decreased from 20.25 per 100,000 in 2015 to 3.44 per 100,000 in 2019. Most of ZIKV infections clustered in Latin America. The proportion of episodes in Central and Tropical Latin America decreased in 2019 with sporadic episodes elsewhere. High Socio-Demographic Index (SDI) regions had more episodes in 2019 than in 2015. Additionally, 15–49 years group had the largest proportion of episodes, females had a higher number of episodes, and a higher incidence rate of 70 plus group was observed in males than females. Certain cities in Europe, North America and Latin America/Caribbean had a high population mobility in ZIKV outbreak areas considered a high risk of imported cases.

**Conclusions:**

ZIKV infection is still a public health threat in Latin America and Caribbean and high SDI regions suffered an increasing trend of ZIKV infection. Interventions such as development of surveillance networks and vector-control should be attached to ZIKV control in these key regions. Reproductive suggestions should be taken to reduce ZIKV-related birth defects for the people of reproductive age who are facing a higher threat of ZIKV infection, especially females. Moreover, surveillance of travellers is needed to reverse the uptrends of travel-related imported ZIKV infection. More studies focusing on ZIKV should be performed to make targeted and effective prevention strategies in the future.

## Author summary

Zika virus (ZIKV) infection is a mosquito-borne illness and has potential result in severe birth effects. Currently, ZIKV is still causing an unprecedented ongoing epidemic in Latin America and threatening North America and potentially the rest of the world. This is the first study to assess the global landscape, long-term trends and regional differences in the incidence of ZIKV infection using the data from Global Burden of Disease (GBD) Study 2019, including the description of ZIKV infection episodes by different sex and by different year group, as well as the relationship between international travellers and imported ZIKV cases. Our study can not only serve as complement to previous studies, but also provide a more comprehensive perspective of global ZIKV infection prevention strategies.

## 1. Introduction

Zika virus (ZIKV) is transmitted primarily by Aedes mosquitoes, biting during the day [1]. ZIKV is circulating in the Americas, South Asia, and the Pacific Islands. Neurological complications and severe fetal outcomes can be caused and ZIKV has become the first major infectious disease linked to human birth defects [2], creating global alarm that the World Health Organization (WHO) declared it the Public Health Emergency of International Concern in 2016 [3]. A long-term response to ZIKV is now being implemented because it is still causing an unprecedented ongoing epidemic in Latin America, threatening North America and potentially the rest of the world.

ZIKV is a positive sense, single-strand ribonucleic acid (RNA) virus [4]. It is a member of the flavivirus genus within the family *Flaviviridae*. Approximately 70 viruses are primarily transmitted by mosquito or tick vectors [5]. ZIKV is likely to have originated in East Africa and subsequently spread to West Africa and then to Asia, resulting in distinct lineages (Nigerian Cluster, MR766 Cluster, and the Asian genotype) [2]. There is strong conservation among all ZIKV strains overall, with less than 12% divergence at the nucleotide level [6]. This is important for diagnostic assays and the development of therapeutics and vaccines.

Compared to other arboviruses such as dengue and chikungunya infections, ZIKV infection involves additional transmission routes. The transmission of ZIKV can simply be divided into mosquito-borne and non-mosquito transmission. It is transmitted to humans primarily through mosquito-borne transmission, with two distinct transmission cycles: (i) a sylvatic cycle, involved in the maintenance of ZIKV between non-human primates and arboreal mosquitoes in forests; and (ii) an urban cycle, involved in the transmission of ZIKV between humans and urban mosquitoes in towns [7]. For nonmosquito transmission, ZIKV can also be passed from the mother to the fetus during pregnancy or sexual contact. In addition to the autochthonous transmission of ZIKV, a potential pandemic threat is currently occurring because there has been an increasing number of travel-related imported ZIKV infection cases in nonendemic countries [4], indicating a large susceptible population.

ZIKV infection is symptomatic in only 20–25% of infected individuals who develop a mild and self-limited illness, with an incubation period of 4–10 days [8]. However, a growing body of evidence suggests that in some severe cases, ZIKV infection can cause neurologic complications and severe fetal outcomes. For neurologic complications, Guillain-Barré syndrome has been observed to have a temporal and geographic relationship with ZIKV outbreaks in the Pacific and the Americas [2]. Meningoencephalitis and acute myelitis complicating ZIKV infection have also been reported. For fetal outcomes, the association between ZIKV infection and cases of microcephaly was first reported in 2015 in Brazil [9]. Vertical transmission has been associated with spontaneous abortion and stillbirth and with congenital malformations in newborns including but not limited to microcephaly [10]. Ocular anomalies have been reported among infants with microcephaly in Brazil [2]. This is one of the reasons that ZIKV infection causes great concern to public health around the world.

Currently, ZIKV transmission persists but has generally been at low levels throughout 2018 to the present. Almost all Latin American and Caribbean countries have already reported active ZIKV circulation [11]. In 2020, a total of 45,848 cases of ZIKV disease were reported in the American region and subregions. Of these, 5,390 (11.8%) were confirmed cases [12]. Moreover, birth defects and long-term neurodevelopmental abnormalities in infants born during the ZIKV epidemic were reported in the Dominican Republic [13], indicating that the burden of ZIKV-related birth defects is still a public threat worldwide.

Due to potential fetal complications, ZIKV infection is still a severe challenge to public health. In this current study, we retrieved detailed information on the incidence of ZIKV infection from the Global Burden of Disease (GBD) study 2019. To understand the global trend and the disease burden of ZIKV infection, we assessed the ZIKV infection incidence from 2011 to 2019 and analyzed it at global, regional and national levels. Our study aimed to understand the global landscape, long-term trends and regional differences in the incidence of ZIKV infection. It can not only serve as a complement to previous studies but also provide a more comprehensive perspective of global ZIKV infection prevention strategies.

## 2. Methods

### 2.1. Data source

This study is a post-hoc analysis of GBD 2019 data, which are available at the Institute of Health Metrics and Evaluation. Number of annual episodes, age-specific incidence rates and age standardized incidence rates (ASRs) of ZIKV infection from 2011 to 2019 were collected, by sex, region, country or territory [14]. Specific methods of GBD 2019 estimation process were described elsewhere [15]. Briefly, data on episodes of acute ZIKV infection and Congenital Zika Syndrome (CZS) come from official reports, primarily from the Pan American Health Organization (PAHO). Incidence of ZIKV infection among pregnant women and CZS was estimated by mixed-effects Poisson regression model [15].

To have a brief understanding of places that are susceptible to imported ZIKV cases, we obtained the World Air Transport Statistics (WATS) from the International Air Transport Association (IATA) for the time period covering entire 2019. IATA data include the origin airport and final destination airport of travellers [16]. We focused on international passengers only and observed the top passenger airport pairs rankings by route area–international and regional traffic.

### 2.2. Regions and demographics

We reported the ZIKV infection in 204 countries and territories, which were divided into five regions by the Socio-Demographic Index (SDI), including low, low-middle, middle, high-middle and high SDI regions. The SDI was developed by GBD researchers and was a composite indicator of the total fertility rate under 25 years, average educational attainment in populations aged 15 years or older, and lag-distributed income per capita. SDI scores were scaled from 0 to 1. An SDI of 0 indicated the lowest income, fewest years of schooling and highest fertility, while an SDI of 1 indicates a theoretical maximum [17]. Meanwhile, the 204 countries and territories were also divided into 21 GBD regions based on their epidemiological homogeneity and geographical contiguity [18]. In this study, we divided into 5 groups: under 5 years, 5 to 14 years, 14 to 49 years, 50 to 69 years, and 70 plus years.

### 2.3. Statistical analysis

The epidemic status of ZIKV infection was shown by age-standardized incidence rate (ASR) with 95% uncertainty intervals (UIs) and absolute number of ZIKV infection episodes. ASRs, standardized by GBD World Standard Population, were used to compare populations with different age structures or for the same population over time in which the age profiles change accordingly [19].

We used the relative changes in ZIKV infection episodes and estimated annual percentage change (EAPC) of ASRs with 95% confidence intervals (CIs) to quantify the ZIKV infection incidence trends. Relative changes in ZIKV infection episodes from 2011 to 2015 and from 2015 to 2019 were defined as $\frac{{Episodes}_{2015}-{Episodes}_{2011}}{{Episodes}_{2011}}\times 100\text{\%}$ and $\frac{{Episodes}_{2019}-{Episodes}_{2015}}{{Episodes}_{2015}}\times 100\text{\%}$ [20]. EAPC is a summary and widely used measure of the ASR trend over a specified time interval. A regression line was fitted to the natural logarithm of the rates, i.e. *y* = *α* + *βx* + *ε*, where *y* = *ln*⁡(*ASR*) and *x* = *calendaryear*. The EAPC was calculated as 100 × (*e*^*β*^ − 1), with its 95%Cis, were obtain to measure the temporal trend of ASRs [21]. The ASR was deemed to be in an increasing trend if the EAPC estimation and the lower boundary of its 95%CI were both >0. In contrast, the ASR showed a decreasing trend if the EAPC estimation and the upper boundary of its 95%CI were both <0 [19].

Global and local mapping were presented using the ArcGIS software. Basemap shapefile’s map content from http://bzdt.ch.mnr.gov.cn/, approval number GS (2016) 1663. All statistics were performed using STATA 16.0.

## 3. Results

### 3.1. Global trends in incidence of ZIKV infection

**Fig 1:**
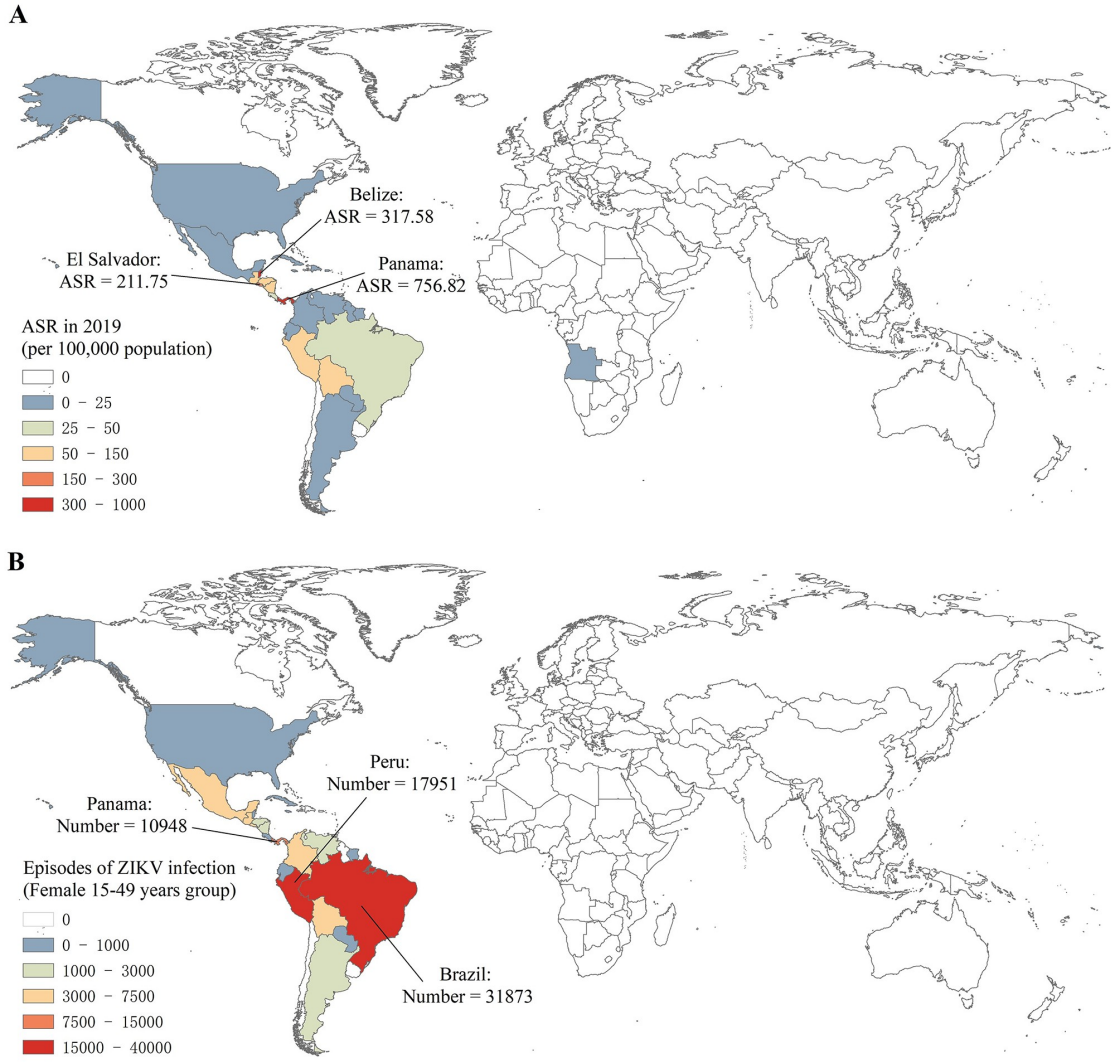
The global incidence of ZIKV infection in 204 countries and territories in 2019. (A) The ASRs of ZIKV infection in 2019; (B) The episodes ZIKV infection of female with age between 15 to 49 in 2019. Global and local mapping were presented using the ArcGIS software. Basemap shapefile’s map content from http://bzdt.ch.mnr.gov.cn/, approval number GS (2016) 1663.

From 2011 to 2019, the highest ASR of ZIKV infection was observed in 2015 (20.25 per 100,000). Globally, the ASR increased by an average of 72.85% (95%CI: 16.47% to 156.53%] per year from 2.09 per 100,000 in 2011 to 20.25 per 100,000 in 2015; subsequently, the ASR decreased with a sizeable fluctuation from 20.25 per 100,000 in 2015 to 3.44 per 100,000 in 2019. In 2019, ZIKV infection still occurred in 28 countries or territories. The highest ASR was observed in Panama (756.82 per 100,000), followed by Belize and EL Salvador (Fig 1A).

The overall number of episodes increased by 912.78% from 150032 in 2011 to 1519501 in 2015 and decreased by 83.32% from 1519501 in 2015 to 268643 in 2019. In 2019, the number of ZIKV infection episodes in Brazil (85109) and Peru (49401) accounted for 31.68% and 18.39% of the world separately (Table 1).

**Table 1:** The number of episodes and age-standardized incidence rates (ASR, per 100 000) of ZIKV infection in 2011, 2015 and 2019, and their temporal trends from 2011 to 2015 and trends from 2015 to 2019.

	Number of episodes					Age-standardized incidence rate (ASR, per 100 000)				
Characteristics	2011 (95% UI)	2015 (95% UI)	2019 (95% UI)	Percentage change 2011–2015 (%)	Percentage change 2015–2019 (%)	2011 (95% UI)	2015 (95% UI)	2019 (95% UI)	EAPC 2011–2015 (95% CI)	EAPC 2015–2019 (95% CI)
Overall	150032 (41722–506407)	1519501 (1090598–2272248)	268643 (203342–366920)	912.78	-82.32	2.09 (0.58–7.07)	20.25 (14.57–30.26)	3.44 (2.60–4.71)	72.85 (16.47 to 156.53)	-32.22 (-58.17 to 9.82)
Male	59492 (16264–199560)	606226 (420047–902472)	109469 (80797–154215)	919.01	-81.94	1.67 (0.46–5.60)	16.15 (11.23–24.14)	2.78 (2.06–3.91)	72.92 (16.51 to 156.63)	-31.92 (-58.53 to 11.76)
Female	90541 (24752–306847)	913274 (628916–1405462)	159174 (119312–220901)	908.69	-82.57	2.55 (0.69–8.64)	24.59 (16.81–37.91)	4.14 (3.09–5.78)	72.80 (16.43 to 156.44)	-32.43 (-57.92 to 8.49)
Low	1822 (475–6370)	18914 (9392–41654)	1566 (677–3817)	938.33	-91.72	0.23 (0.06–0.80)	2.14 (1.02–4.81)	0.16 (0.07–0.40)	71.49 (15.41 to 154.83)	-48.77 (-54.57 to -42.22)
Low-middle	69259 (17891–233286)	703124 (461137–1167148)	93841 (67184–139280)	915.21	-86.65	4.45 (1.16–15.03)	42.41 (28.06–70.47)	5.30 (3.81–7.89)	72.27 (16.03 to 155.77)	-36.45 (-64.46 to 13.65)
Middle	52742 (13882–182011)	536098 (298146–1065908)	138616 (92246–231357)	916.46	-74.14	2.33 (0.61–8.03)	22.72 (12.50–45.33)	5.70 (3.76–9.48)	73.29 (16.90 to 156.87)	-25.46 (-55.96 to 26.15)
High-middle	26155 (6763–89126)	260604 (124265–595311)	33606 (17501–72743)	896.40	-87.10	1.89 (0.49–6.45)	18.42 (8.83–41.29)	2.35 (1.23–5.17)	73.21(16.57 to 157.38)	-41.14 (-55.72 to -21.77)
High	9 (2–30)	288 (121–680)	887 (398–2458)	3152.54	207.62	0	0.03 (0.01–0.07)	0.09 (0.04–0.25)	131.42 (63.92 to 226.71)	30.20 (-85.44 to 1064.01)
High-income Asia Pacific	0	0	0	-	-	0	0	0	0	4.15 (-46.12 to 101.32)
South Asia	0	0	0	-	-	0	0	0	0	3.92 (-84.02 to 575.67)
Southeast Asia	0	0	0	-	-	0	0	0	0	3.95 (-46.98 to 103.82)
Caribbean	1370 (364–4624)	14017 (7537–31092)	3729 (2068–7213)	923.36	-73.40	3.06 (0.81–10.32)	30.36 (16.41–68.03)	7.76 (4.29–14.94)	73.94 (17.15 to 158.27)	-22.70 (-74.27 to 132.21)
Andean Latin America	23 (6–81)	837 (298–2091)	62636 (28432–147666)	3506.40	7382.85	0.04 (0.01–0.15)	1.40 (0.50–3.49)	97.92 (44.25–231.31)	134.62 (66.18 to 231.23)	144.85(-79.09 to 2766.63)
Central Latin America	75078 (19531–253970)	754231 (441292–1396118)	109761 (76296–167365)	904.59	-85.45	32.26 (8.40–109.34)	308.33 (179.90–567.59)	43.33 (30.11–66.18)	72.37 (16.14 to 155.83)	-38.96 (-47.30 to -29.30)
Southern Latin America	0	0	4802 (1324–13383)	0.00	-	0	0	7.11 (1.97–19.51)	0	55.72 (-59.57 to 499.84)
Tropical Latin America	73559 (20066–248772)	750390 (515457–1229968)	86827 (61394–131710)	920.12	-88.43	34.75 (9.52–117.57)	338.91 (233.29–557.00)	37.79 (26.79–57.23)	73.25 (16.78 to 157.02)	-43.04 (-51.42 to -33.21)
High-income North America	0	0	871 (391–2448)	0.00	-	0	0	0.24 (0.11–0.69)	0	-21.31 (-60.08 to 55.11)
Oceania	2 (0–10)	25 (0–97)	0	984.49	-100.00	0.02 (0.00–0.09)	0.21 (0.00–0.80)	0	73.61 (16.81 to 158.03)	40.91 (-60.53 to 403.10)
Central Sub-Saharan Africa	0	0	16 (4–44)	0.00	-	0	0	0.01 (0.00–0.02)	0	-60.31 (-90.27 to 61.96)

Abbreviations: ASR, age-standardized incident rate; CI, confidence interval; EAPC, estimated annual percentage change; GBD, Global Burden of Disease; ZIKV, Zika virus; UI, uncertainty interval.

Comments:

High-income Asia pacific: ZIKV outbreaks occurred in Singapore from 2016 to 2018, with the highest episodes observed in 2017.

South Asia: ZIKV outbreaks occurred in Thailand and Vietnam from 2016 to 2018, with the highest episodes observed in 2017.

Southeast Asia: ZIKV outbreaks occurred in India from 2016 to 2018, with the highest episodes observed in 2017.

Andean Latin America: ZIKV outbreaks occurred in Peru from 2016 to 2019, with the highest episodes observed in 2017. An increasing trend was observed in Ecuador from 2011 to 2017. The highest number of episodes was observed in Bolivia in 2018, while a similar number was reported in 2017 and 2019.

Southern Latin America: ZIKV outbreaks occurred in Argentina from 2016 to 2019, with the highest episodes observed in 2017. ZIKV infection is still being reported.

High-income North America: ZIKV outbreaks occurred in the United States of America from 2016 to 2019, with the highest episodes observed in 2017. ZIKV infection is still being reported.

Oceania: ZIKV outbreaks occurred in American Samoa from 2016 to 2018, with the highest episodes observed in 2017. ZIKV infection was reported in Papua New Guinea in 2016.

### 3.2. Differences in incidence of ZIKV infection by 21 GBD regions

**Fig 2:**
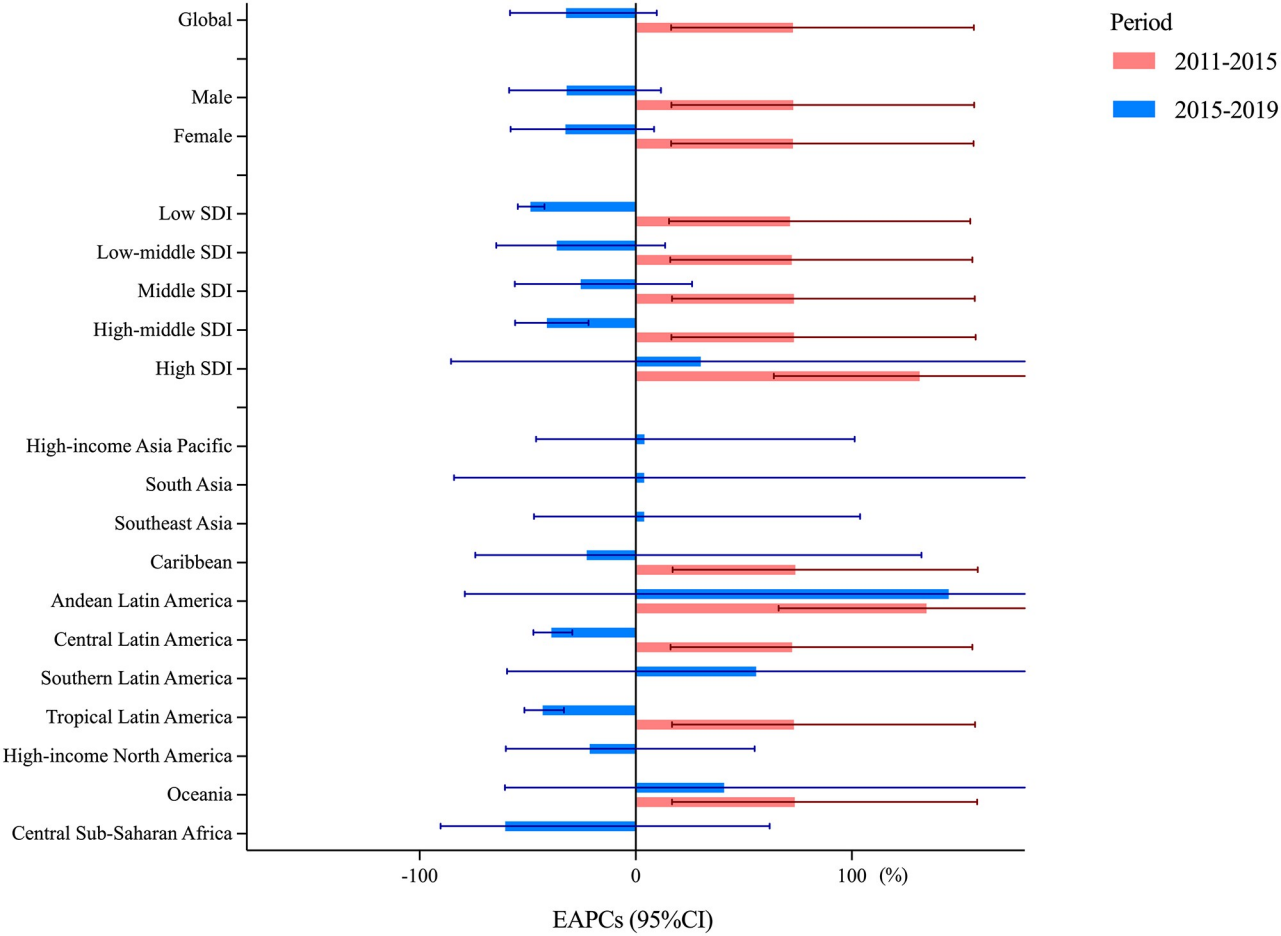
The EAPCs of ZIKV infection ASRs from 2011 to 2015, and from 2015 to 2019 by regions. EAPC, estimated annual percentage change; ASR, age-standardized incidence rate; CI, confidence interval; SDI, Socio-demographic Index.

In 2019, ZIKV infection occurred in 7 GBD regions and most of them clustered in Latin America. Andean Latin America suffered the highest threat of ZIKV infection with an ASR of 98 per 100,000, followed by Central and Tropical Latin America with an ASR over 40 per 100,000 (Table 1 and Fig 1A). From 2011 to 2015, increase in ASR were observed in all GBD regions, especially in Central Latin America (EAPC = 72.37; 95%CI: 16.14 to 155.83) and Tropical Latin America (EAPC = 73.25; 16.78 to 157.02). From 2015 to 2019, a decreasing trend was observed in most of GBD regions (Fig 2).

**Fig 3:**
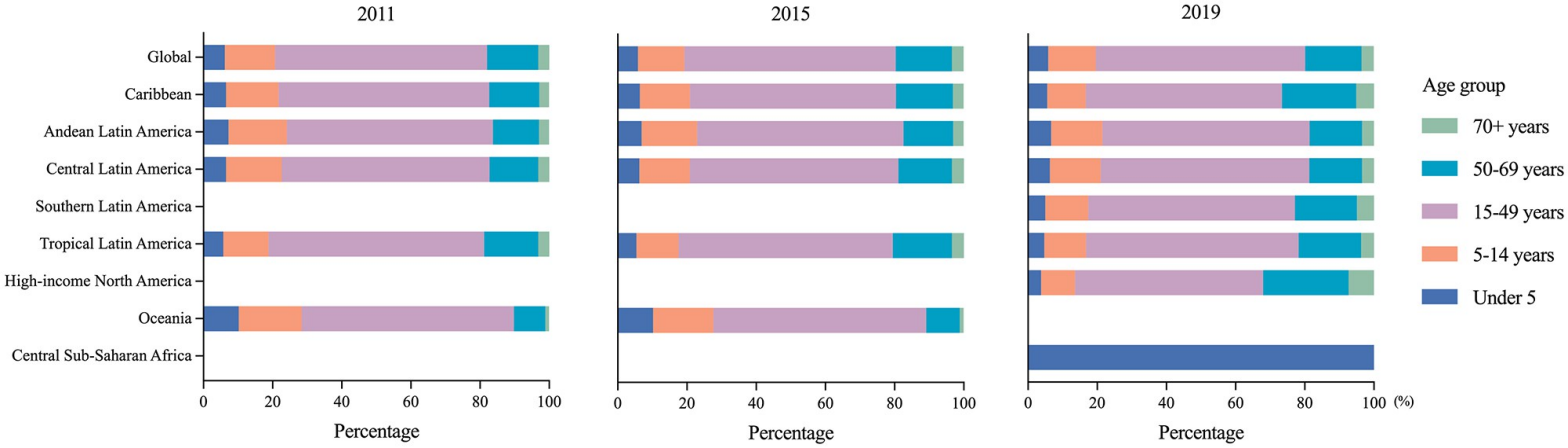
Age group distribution of ZIKV infection episodes by GBD region in 2011, 2015 and 2019.

Over 99% episodes were observed in Central and Tropical Latin America in 2011 and 2015; however, only 73.18% episodes were observed in Central and Tropical Latin America with sporadic episodes elsewhere in 2019. Age group proportions of ZIKV infection episodes in 2011, 2015 and 2019 were presented (Fig 3). Approximately 60% of the episodes were 15–49 years people in 2011–2019.

### 3.3. Differences in incidence of ZIKV infection by five SDI regions

The highest ASR was observed in middle SDI regions (5.70 per 100,000), with the largest number of ZIKV infection episodes (138616) in 2019 (Table 1). From 2011 to 2015, the ASR increased in all SDI regions, by the most in high SDI regions (EAPC = 131.42; 63.92 to 226.71). From 2015 to 2019, the ASR decreased in 4 other SDI regions in addition to high SDI.

**Fig 4:**
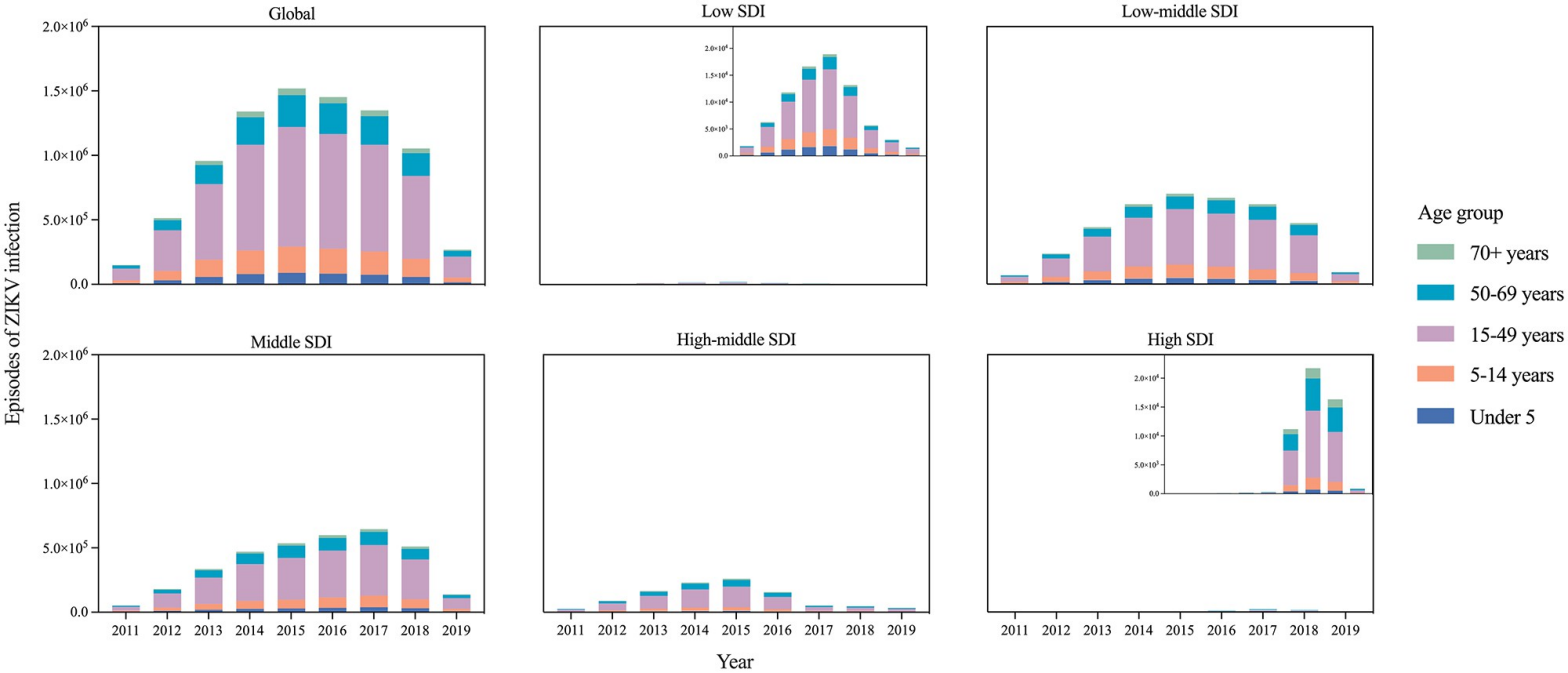
The episodes of ZIKV infection by age group, and by SDI region, from 2011–2019. SDI: Socio-demographic Index.

In low-middle, middle and high-middle SDI regions, there were significantly more ZIKV infection episodes. In high SDI regions, the number of ZIKV infection episodes increased remarkably (207.62%) in 2015–2019. In all the regions, the proportion of ZIKV infection episodes was stable in all age groups in 2011, 2015 and 2019 (Fig 4). The proportion of ZIKV infection was highest in 15–49 years group, followed by the 50–69 years group, in addition to the low SDI regions.

### 3.4. Differences in incidence of ZIKV infection by sex and age

**Fig 5:**
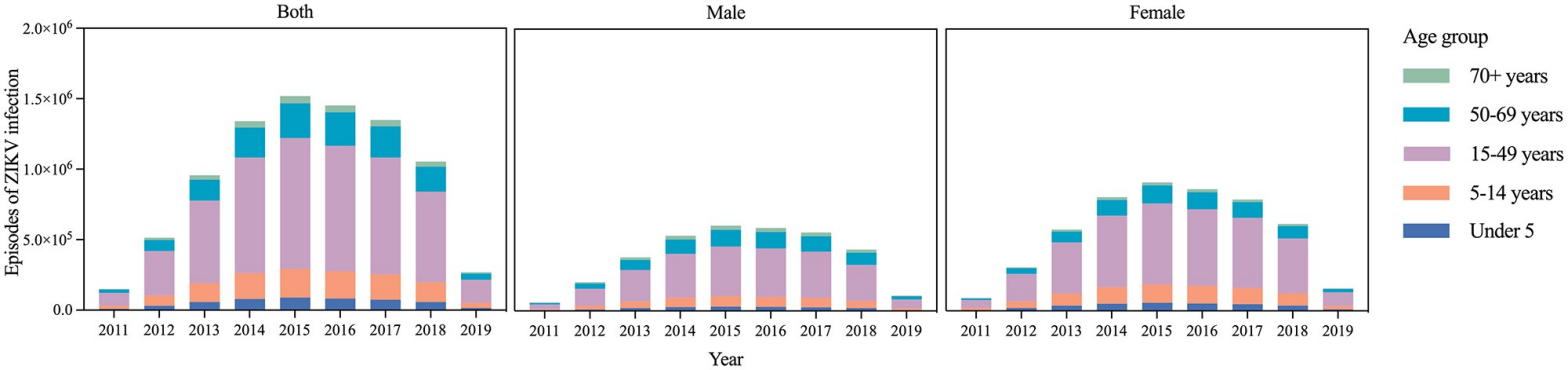
The episodes of ZIKV infection by age group, and by sex, from 2011–2019.

Higher number of ZIKV infection episodes was observed in females each year (Table 1), with a higher number of females (59.25%) than males (40.75%) in 2019. In 2019, the number of episodes in Brazil (20.02%) and Peru (11.21%) accounted a large proportion of the world female 15–49 years group (Fig 1B). The ASR increased in 2011–2015 and decreased in 2015–2019 in both sexes (Table 1 and Fig 5). The proportions of ZIKV episodes by age group at the sex level in 2011–2019 were presented (Fig 5). Both females and males had approximately 60% of the episodes in 15–49 years group. Under 50 years group had a higher proportion of episodes among females, while males had a higher proportion in over 50 plus years group. Moreover, the male group had the highest incidence rate of ZIKV infection in 50–69 group and a higher incidence rate than the female group in 70 plus group (S1 Fig).

### 3.5. International travel numbers of ZIKV related regions

According to the WATS of IATA in 2019, 3 route areas were associated with ZIKV outbreak areas (Latin America/Caribbean): Europe-Latin America/Caribbean, Latin America/Caribbean-North America, and Within Latin America/Caribbean. Cities were considered to have a high risk of imported ZIKA cases in our study, if they were the top 10 airport pairs in the 3 route areas, indicating the highest population mobility.

Among the 3 routes, route Latin America/Caribbean-North America had the highest passenger carried number. The United States and Canada had a large population of international travellers from Latin America/Caribbean in 2019, with almost 7 million people in the top 10 airport pairs. Miami, New York, Los Angeles and Toronto had major final destination airports, which might suffer a larger threat of imported ZIKV cases. For the Europe, 4 countries (France, Spain, the United Kingdom and the Netherlands) involved more air traffic with ZIKV outbreak areas. Spain, especially Madrid and Barcelona, suffered the severest threat of imported ZIKV cases with a high international traveller number. For the route Within Latin America/Caribbean, there were many interactive flights among Chile, Brazil, Argentina and Peru. Additionally, the airport pairs of Buenos Aires-Santiago de Chile and Buenos Aires-Sao Paulo had 42.6% and 63.0% growth than 2018, indicating a great increasing risk of ZIKV infection in these cities should pay attention to (S1 Table).

## 4. Discussion

To our knowledge, this is the first study to assess the global landscape, long-term trends and regional differences in incidence of ZIKV infection using GBD study data. Description of ZIKV infection episodes by sex and by different year group and the relationship between international travellers and imported ZIKV cases were also mentioned. Globally, the overall ASR of ZIKV infection episodes showed an increasing trend from 2011 to 2015 and the ASR decreased with a sizeable fluctuation from 2015 to 2019. Latin American and Caribbean countries suffered a high threat of ZIKV infection. Meanwhile, the high SDI regions had the most obvious increase from 2011 to 2015 and a different trend was observed from the global downward trend from 2015 to 2019. The largest proportion was observed in 15–49 years group. Additionally, females had more episodes than males; however, there was a higher incidence rate of males in 70 plus group. Moreover, many cities in Europe, North America and Latin America/Caribbean had a high population mobility in the ZIKV outbreak area, which is considered a high risk of imported ZIKA cases. Due to the severe birth defects associated with ZIKV infection, prevention is necessary and is the most effective approach against ZIKV infection. More efforts should be made to eliminate ZIKV infection globally.

Comprehensive observation of ZIKV infection in various regions of the world revealed that ZIKV infection emerged in 2015 with the largest episodes and ASR. Previous studies showed the same situation as our study [22] with 33 countries reporting autochthonous transmission of ZIKV infection and an increase in the incidence of cases of microcephaly and/or Guillain-Barré syndrome in 2015 [23]. In 2016, *Zika Strategic Response Plan* was published, encouraging the development of surveillance networks, diagnostics, risk communication strategies, and vector-control measures [24,25]. Now, ZIKV transmission persists but has generally been at low levels throughout 2018 to the present, indicating that the strategies had been well used in recent years. Previous studies were consistent with our study [11,26].

Currently, ZIKV infection burden still occurs in Latin American and Caribbean countries with sporadic episodes elsewhere. Almost all Latin American and Caribbean countries have already reported active ZIKV circulation [11] [27], which supported our results. Low incidence was observed in Africa and Asia, probably due to the likelihood of underdiagnosis, lower risk of ZIKV introduction and immune issues (e.g., due to previous ZIKV infection or cross-protection conferred by previous dengue infections) [28,29]. However, there is still a potential risk of ZIKV epidemics. For instance, low population-level exposure to ZIKV had reported in African regions [30]. Uttar Pradesh state of India also confirmed one hundred cases of ZIKV disease during October-November 2021 [31]. The transmitters of ZIKV, *Aedes aegypti* and *Aedes albopictus* mainly [32], which are widely distributed in the tropical and subtropical regions. ZIKV transmission has obvious regional and seasonal heterogeneity and is related to environmental changes, especially climate change [33,34]. In addition, a study predicted that the potential for ZIKV transmission risk is simulated to increase over southern and Eastern Europe, northern America and temperate regions of Asia (northern China, southern Japan) in future climate scenarios [35]. More attention should be given to potential ZIKV outbreak areas. Vector-based interventions are the principal methods available for reducing the public health burden of most mosquito-borne diseases [36].

The incidence of ZIKV infection is closely related to socioeconomic development. Middle SDI regions were observed to have the largest number of ZIKV infection episodes. Meanwhile, few ZIKV infections occurred in low SDI regions, probably due to a lack of disease awareness, poor access to diagnostics, low accuracy of detection and underreporting [2,37]. ZIKV infection remains a public health threat, especially for developing nations where scarcity of resources and unhygienic conditions in hospitals already limits access to timely prevention [38]. Thus, it is worth calling for increasing awareness, and better testing systems and timely surveillance systems of ZIKV infection. ZIKV infection in high SDI regions was worth concerning in recent years due to the largest growth from 2011 to 2015 and an increased ZIKV infection episode from 2015 to 2019. Changing lifestyles for globalization and urbanization may be primary risk factors in high SDI regions. Increased connectivity via air travel can facilitate the geographic spread of infectious diseases [39]. Not only travellers are at individual risk, but they contribute substantially to the rapid spread [40] by returning to countries where competent vectors are present, thereby providing a source for autochthonous transmission [40]. A study predicted that a large portion of tropical and subtropical regions globally have suitable environmental conditions with over 2.17 billion people inhabiting these areas [41], raised concerns about the high risk of introducing and establishing new autochthonous transmission in these areas [4]. These travel-related ZIKV infections can be reduced by the following: a) travellers should discuss their travel plans with their healthcare providers to ensure that prevention measures are taken [42,43]; b) ZIKV infection should remain a consideration for travellers returning from the ZIKV outbreak area [42]; and c) avoidance of the primarily daytime-biting *Aedes* mosquitos both during and after travel [43].

There is strong relationship between imported cases and international travel. Thus, the air transport information of international travellers who went to the area associated with ZIKV outbreaks may provide a forecast and warning of ZIKV infection. Moreover, mobility patterns and travel volumes can help to identify the most likely origin of importation and also in predicting further propagation [44]. Since early 2015, there have been an increasing number of travel-related imported ZIKV cases in nonendemic countries [4]. According to our results, Europe and North America had a higher threat of imported ZIKV cases. For Europe, the risk of ZIKV infection in Spain, France and the Netherlands wes specifically mentioned. The European Centre for Disease Prevention and Control (ECDC) reported that 92% of cases of ZIKV disease with known importation status were from returning travellers, and Spain, France and Germany were the top three countries with the largest number of episodes in 2019 [45]. A total of 56.8% of ZIKV infection in EU (European Union)/EEA (The European Economic Area) travellers originating from the Caribbean was reported in 2018 [46], supporting our results. For North America, the United States as a single country was a primary exported case destination outside Latin America as it received the highest travel volumes [47]. For instance, a total of 588 travel-associated ZIKV infection episodes were reported in California during November 2015-September 2017 with most case-patients reporting travel to Mexico and Central America [48]. If possible, countries with both warning and stable coping capacity to existing and novel pathogens should pay attention to surveillance of travellers and improve the capacity to detect and respond to infectious disease threats that emerge within their borders [39].

Most ZIKV infection episodes were between 15 to 49 years old, including the reproductive age. More episodes occurred in females. It has been confirmed that ZIKV infection during pregnancy is deleterious to the fetus [49] and the clinical evidence of microcephaly and Guillain-Barré syndrome has been confirmed [50]. A study showed that pregnant women returning from ZIKV affected areas are at risk of acquiring the infection and presenting harmful consequences on their offspring [51]. Therefore, reproductive age people should make sustained efforts to reduce ZIKV-related birth defects: a) avoiding unnecessary travel to ZIKV outbreak area [2]; b) preconception counseling for ZIKV infection risk, safer sex counseling and contraception [52,53]; c) using condoms correctly and consistently or abstain from having sex during 3 months after known or presumptive infection [53,54]; d) pregnant women or whose sexual partner has traveled to countries with local mosquito-borne transmission should be tested for ZIKV [52]; e) pregnant women who have confirmed a possible or presumptive infection should receive serial fetal ultrasounds [52]. Additionally, direct human-to-human transmission of ZIKV occurs not only sexually but perinatally and through breastfeeding or blood transfusion [4], further increasing the disease burden of reproductive age females. Effective prevention of ZIKV infection should be performed at both the personal and integrated levels.

In addition, the increasing number of ZIKV infection episodes that occurred in males more than 50-year-old caused a concern of male ZIKV infection. There is no evidence for this conclusion now, but this may be a new high-risk group for ZIKV infection. A study showed that ZIKV is detectable in human semen during acute infection and may remain in the genital tract and facilitate long-term transmission [55]. In addition, an 81-year-old man infected with ZIKV developed meningoencephalitis, indicating a possible association with other neurological complications in elderly individuals [56]. Further research is needed on the abnormal incidence in males in this age group.

However, it is a great challenge to prevent infections in both travellers and residents of endemic settings because no vaccines or antiviral treatments have been approved to cure ZIKV infection currently [23]. The considerable cross-reactivity of flavivirus antibodies presents major challenges for the interpretation of serologic test results [2], and the identification test is labor-intensive and costly. Moreover, the simultaneous co-circulation of dengue virus (DENV), chikungunya virus (CHIKV), ZIKV and their co-infections has been reported [57]. Thus, prevention is the best method of protection against ZIKV infection at present.

The GBD study provides a better understanding of the incidence of ZIKV infection that targeted global prevention strategies may be facilitated further. There are some limitations of this study. First, the quality and quantity of data may be affected by diagnostics and the underreporting of episodes, while the lacking national systematic surveillance of some countries may be a margin of bias. Second, we only use the air freight information which may not represent the overall situation of travellers. Third, due to the sizeable fluctuations in the epidemiology of ZIKV infection in different countries and territories, our method may have limitations in describing the overall trend causing the large confidence intervals of EAPC in some regions. Thus, we combed the outbreak of these regions and explained the trend in specific countries to cover this question.

In summary, ZIKV infection is still a public health threat in Latin America and Caribbean, and high SDI regions suffered an increasing trend of ZIKV infection; therefore, interventions such as development of surveillance networks and vector-control measures should be attached to ZIKV control in these key regions. Reproductive suggestions at both the personal and integrated levels should be taken to reduce ZIKV-related birth defects for the people of reproductive age who are facing a higher threat of ZIKV infection, especially for females. Moreover, due to the changing lifestyle for globalization and urbanization, surveillance of travellers is needed to reverse the uptrends of travel-related imported ZIKV infection in Europe, North America and Latin America/Caribbean. Currently, it is a welcome sign that the reported cases and birth defects are decreasing, and should not be a cause for relaxing the vigilance of ZIKV infection. More studies focusing on ZIKV should be performed to make targeted and effective prevention strategies in the future.

## Supporting information

S1 Fig The number of ZIKV incident rate by age group, and by sex, from 2011–2019. (TIF)

S1 Table The passenger carried number of top passenger airport pairs by route area associated with ZIKV outbreak in 2019. (DOCX)

S1 Data Excel spreadsheet containing, in separate sheets, the underlying numerical data and statistical analysis for Figs 1A and 1B, 2, 3, 4, 5 and S1 Fig. (XLSX)
