# Supplementary figures and images for: The global trends and regional differences in incidence of Zika virus infection and implications for Zika virus infection prevention
Source: PLoS Negl Trop Dis. 2022 Oct 21;16(10):e0010812. doi: 10.1371/journal.pntd.0010812 (PMC9586358; doi:10.1371/journal.pntd.0010812)

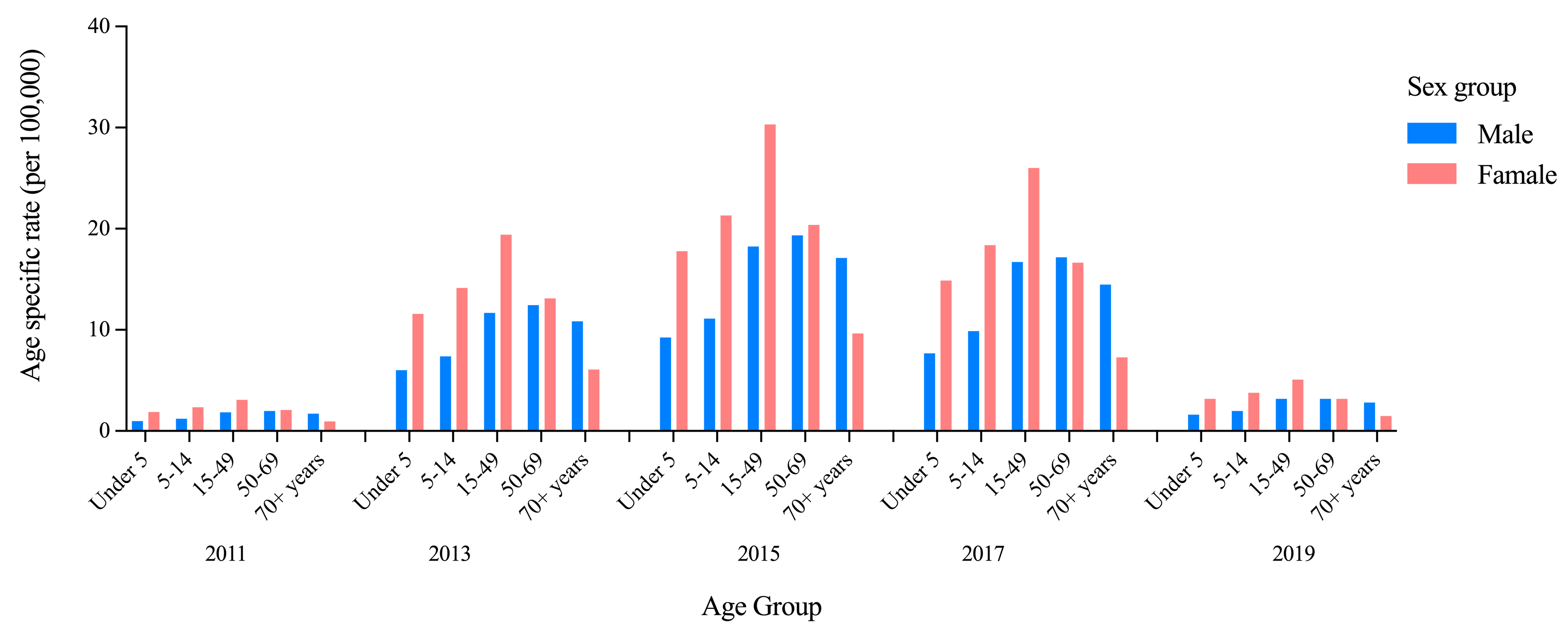

Supplement: S1 Fig — (TIF) [file pntd.0010812.s001.tif]
