# Supplementary material for: The global trends and regional differences in incidence of Zika virus infection and implications for Zika virus infection prevention
Source: PLoS Negl Trop Dis. 2022 Oct 21;16(10):e0010812. doi: 10.1371/journal.pntd.0010812 (PMC9586358; doi:10.1371/journal.pntd.0010812)
Supplement: S1 Table — (DOCX) [file pntd.0010812.s002.docx]

Table S1 The passenger carried number of top passenger airport pairs by route area associated with ZIKV outbreak in 2019.

|  | Airport 1 |  | Airport 2 |  |  |  |
| --- | --- | --- | --- | --- | --- | --- |
| Route Area | **City** | **Country** | **City** | **Country** | **Passenger carried number** | **Growth in 2019 over 2018 (%)** |
| Europe – Latin  America/Caribbean | Paris | France | Pointe-a-Pitre | Guadeloupe | 1210246 | 21.4 |
|  | Fort de France | Martinique | Paris | France | 1030247 | 16.1 |
|  | Buenos Aires | Argentina | Madrid | Spain | 575383 | 6.0 |
|  | Barcelona | Spain | Buenos Aires | Argentina | 435367 | 19.6 |
|  | Bridgetown | Barbados | London | The United Kingdom | 408341 | 7.3 |
|  | Amsterdam | Netherlands | Curacao | Netherlands | 396239 | -3.6 |
|  | Madrid | Spain | Mexico City | Mexico | 396137 | 4.7 |
|  | Bogota | Colombia | Madrid | Spain | 395664 | 21.4 |
|  | Lima | Republic of Peru | Madrid | Spain | 377096 | 19.1 |
|  | Amsterdam | Netherlands | Paramaribo | The Republic of Suriname | 357216 | 19.1 |
| Latin America/Caribbean - North America | Buenos Aires | Argentina | Miami | The United States | 824017 | -10.2 |
|  | Cancun | Mexico | Toronto | Canada | 802605 | 11.8 |
|  | Guadalajara | Mexico | Los Angeles | The United States | 790353 | 0.3 |
|  | Sao Paulo | Brazil | Miami | The United States | 690743 | 4.8 |
|  | New York | The United States | Mexico City | Mexico | 689506 | -1.2 |
|  | Los Angeles | The United States | Mexico City | Mexico | 658668 | -19.6 |
|  | New York | The United States | Santiago | Chile | 629541 | 0 |
|  | Mexico City | Mexico | Miami | The United States | 564447 | 18.2 |
|  | New York | The United States | Santo Domingo | The Dominican Republic | 564234 | -0.1 |
|  | Punta Cana | The Dominican Republic | Toronto | Canada | 545798 | 3.5 |
| Within Latin  America/Caribbean | Lima | Republic of Peru | Santiago de Chile | Chile | 981935 | 3.3 |
|  | Buenos Aires | Argentina | Santiago de Chile | Chile | 853378 | 42.6 |
|  | Buenos Aires | Argentina | Sao Paul | Brazil | 683581 | 63 |
|  | Buenos Aires | Argentina | Rio de Janeiro | Brazil | 585001 | 0.1 |
|  | Rio de Janeiro | Brazil | Santiago de Chile | Chile | 550389 | -5.8 |
|  | Sao Paulo | Brazil | Santiago de Chile | Chile | 504993 | -11.5 |
|  | Bogota | Colombia | Lima | Republic of Peru | 407251 | 11.3 |
|  | Buenos Aires | Argentina | Lima | Republic of Peru | 361886 | -4.6 |
|  | Fort de France | Martinique | Pointe-a-Pitre | Guadeloupe | 351993 | -7.5 |
|  | Cancun | Mexico | Havana | Cuba | 345394 | -5.2 |
